# Identification of efflux inhibitors through a drug repurposing strategy in *Candida albicans*

**DOI:** 10.3389/fcimb.2026.1798450

**Published:** 2026-05-18

**Authors:** Débora Marques, Sofia Santos Costa, Liliana Rodrigues

**Affiliations:** Global Health and Tropical Medicine, GHTM, LA-REAL, Instituto de Higiene e Medicina Tropical, IHMT, Universidade NOVA de Lisboa, Lisboa, Portugal

**Keywords:** *Candida albicans*, drug repurposing, efflux inhibitors, efflux pumps, phenotypic screening

## Abstract

**Introduction:**

*Candida albicans* is a major cause of nosocomial fungal infections, often associated with high morbidity and mortality. The rising resistance to antifungal drugs, particularly azoles, underscores the need for new therapeutic strategies. Among resistance mechanisms, alterations in drug targets and overexpression of efflux pump genes play key roles. Efflux inhibitors can enhance the intracellular drug accumulation, thereby restoring antifungal efficacy and preventing resistance development. Drug repurposing offers a cost-effective and time-efficient alternative for identifying new drugs, including efflux inhibitors. In this study, we used an *in silico* drug repurposing approach to identify approved drugs associated with membrane transport functions and evaluate their efflux-modulating potential *in vitro*.

**Methods:**

Using the DrugBank database, we screened predicted membrane transport proteins in *C. albicans* and selected those with homology to known drug targets. A subset of drugs, representative of each chemical group, was selected for *in vitro* screening against the reference strain *C. albicans* ATCC 90028. We further evaluated the potential adjuvant effect of these drugs on the activity of fluconazole. Finally, the efflux inhibitory effect of the candidate drugs was assessed by real-time fluorometric detection of ethidium bromide accumulation.

**Results:**

A total of 245 predicted membrane transport proteins were screened, 51 of which showed homology to known drug targets and were associated with 777 drugs. A subset of 59 drugs was selected for screening. The drug miltefosine showed the lowest minimum inhibitory concentration (MIC; 2 mg/L), while amlodipine and procainamide demonstrated an adjuvant effect by decreasing the MIC of fluconazole by at least four-fold. Fluorometric assays revealed amlodipine, fluvoxamine and fluoxetine as potential efflux inhibitors in *C. albicans*.

**Discussion:**

These findings highlight the potential of these drugs to contribute to the research and development of new therapeutic alternatives aimed at combating antifungal resistance.

## Introduction

Invasive fungal infections, including candidemia, are severe conditions that can be fatal without accurate diagnosis and treatment. Currently, over 200 species of the *Candida* genus have been identified, with *Candida albicans* being one of the most frequent pathogens, affecting at least 10% of patients in intensive care units worldwide, with a high mortality rate (Bassetti et al, 2014, Bassetti et al., 2019; Lass-Flörl and Steixner, 2023; Salmanton-García et al, 2024). In Europe, approximately 79 new cases are reported daily, and it is estimated that 37% of these patients will have a fatal outcome within 30 days of diagnosis, reflecting a high monthly mortality rate associated with candidemia (Hoenigl et al, 2023).

This high morbidity and mortality are largely attributed to the increasing resistance to antifungal agents, a problem further exacerbated by the limited number of antifungal classes available, which often leads to the misuse and overuse of conventional therapies (Arastehfar et al, 2020). Azole resistance, which has seen a notable rise in recent years, is often multifactorial, involving mutations and/or overexpression of genes encoding target proteins, as well as the activity of efflux pumps, frequently arising after prolonged antifungal therapy (Arendrup, 2013; Pristov and Ghannoum, 2019).

Among the primary mechanisms contributing to azole resistance in clinical isolates of *C. albicans* are efflux systems, which are categorized into two main classes: ATP-binding cassette (ABC) transporters, represented mainly by CDR1 and CDR2, and the major facilitator superfamily (MFS) transporters, represented by MDR1 (Prasad et al, 2006; Nishimoto et al, 2020).

Due to the limited range of available antifungal agents, there is an urgent need to develop new antifungal therapies, with the inhibition of efflux pumps being recognized as a critical strategy to counteract antifungal resistance (Vega-Chacón et al, 2021). Efforts to overcome efflux-mediated antifungal resistance include the direct inhibition of efflux pumps and drug repurposing strategies, which have emerged as viable approaches to reduce both the time and cost of drug development, while uncovering new therapeutic applications for already approved drugs beyond their original indications (Pushpakom et al, 2019).

Recent studies have shown that certain drugs, such as verapamil, ribavirin, curcumin, and fluoxetine, may serve as potent efflux inhibitors and display synergistic effects when combined with fluconazole (FLU) (Oliveira et al, 2014; Vega-Chacón et al, 2021; Zhang et al, 2021; Lee et al, 2022). However, there are still limited studies on drug repurposing in *C. albicans*. Therefore, this study aims to apply an *in silico* drug repurposing approach to identify approved drugs with potential efflux inhibitory activity. The approach involves the creation of a virtual library of membrane transport proteins in *C. albicans*, enabling the identification of approved drugs targeting homologous proteins of predicted transporter-related targets. Rather than aiming to validate specific transporter-drug interactions, this study focuses on the functional identification of efflux-modulating phenotypes using a combination of *in silico* prioritization and *in vitro* assays.

## Materials and methods

### Reagents

Glucose, dimethyl sulfoxide (DMSO) and sulfinpyrazone were purchased from Merck (Darmstadt, Germany). Atenolol, azelastine, buspirone, citalopram, diltiazem, flurbiprofen, fluvoxamine, lovastatin, meloxicam, metoclopramide, mycophenolate mofetil, nifedipine, nizatidine, phenytoin, probenecid, risperidone, sertraline, sunitinib and valsartan were purchased from Thermo Fisher Scientific (Massachusetts, USA). Albendazole, anastrozole, benzocaine, dipyridamole, doxepin, enalapril, fluoxetine, gliquidone, imipramine, istradefylline, lansoprazole, miltefosine, olanzapine, ranitidine, repaglinide, tinidazole, tolbutamide and zolmitriptan were purchased from Tokyo Chemical Industry Co., Ltd. (Tokyo, Japan). Avanafil, bisoprolol, diosmin, hydrochlorothiazide, ketoprofen and streptozotocin were purchased from Cayman Chemical (Michigan, USA). Phosphate buffered saline (PBS), Roswell Park Memorial Institute (RPMI-1640), acebutolol, amlodipine, atovaquone, chloroquine, chlorpromazine, desipramine, doxorubicin, duloxetine, FLU, metformin, procainamide, reserpine, sulpiride, topiramate, valproic acid and verapamil were purchased from Sigma-Aldrich Inc. (St. Louis, MO, USA). Bacto™ Brain-heart infusion (BHI) was purchased from BD Difco™ (Sparks, USA.), malt extract from Liofilchem^®^ S.r.l. (Roseto degli Abruzzi, Italy) and ethidium bromide (EtBr) from Panreac AppliChem (Darmstadt, Germany).

All stock solutions were prepared on the day of the experiment in DMSO, except for bisoprolol, chloroquine, desipramine, diltiazem, enalapril, imipramine, metoclopramide, streptozotocin, verapamil and EtBr, which were prepared in sterile deionized water.

### *Candida albicans* growth conditions

A reference strain of *C. albicans* (ATCC^®^ 90028™; American Type Culture Collection, Rockville, MD, USA) was used for the *in vitro* testing of candidate drugs. The strain was stored at -80 °C and recovered in BHI broth supplemented with malt extract (25 g/L), followed by incubation at 35 °C until growth was observed. Subsequently, 0.1 mL of culture were transferred to solid BHI medium and incubated for 24 hours at 35 °C. For all experimental procedures, a 24-hour *C. albicans* culture, incubated at 35 °C in either solid or liquid BHI medium (depending on the specific protocol), was consistently used.

### *In silico* drug repurposing

A stepwise *in silico* screening strategy was used to identify clinically approved drugs potentially interacting with proteins associated with membrane transport in *C. albi*cans. The construction of a virtual library of approved drugs with potential activity against *C. albicans* began with the compilation of membrane transport proteins from the annotated genome of the *C. albicans* SC5314/ATCC MYA-2876 strain, available in the Kyoto Encyclopedia of Genes and Genomes (KEGG; genome ID: T00189) and UniProt (proteome ID: UP000000559) databases (Kanehisa, 2019; UniProt Consortium, 2023). In the case of KEGG, the strategy was as follows: search in the categories “transporters”, “and “membrane transport”. The search results obtained with KEGG were cross-checked and complemented using Uniprot, with searches conducted within the categories “transporter activity”, and “transmembrane transport”. The target name, the respective amino acid sequence in FASTA format, and the associated biological process were compiled into an Excel database for subsequent analysis.

In the following step, the amino acid sequences corresponding to each potential target were used as queries in homology searches against the DrugBank database targets using BLAST-based similarity searches (Wishart et al, 2018). The DrugBank search is based on the principle of homology, wherein similar targets are likely to interact with similar drugs (Neves et al, 2015; Rodrigues et al, 2022). Accordingly, each query (query = protein of *C. albicans*) involved comparing the amino acid sequence of the *C. albicans* target protein against all known targets in the DrugBank database to identify homologous proteins associated with approved drugs. Two inclusion criteria were applied during the search: only hits with an expected value (e-value) ≤ 10^–20^ were retained to ensure high-confidence homology relationships; and only targets associated with drugs approved for clinical use in humans were selected. To refine the list of identified compounds, drugs classified as nutraceuticals, antibiotics, antivirals, hormones, steroids, or those withdrawn from the market were excluded. Finally, the remaining drugs were categorized according to their chemical superclass, as defined by DrugBank.

### Determination of minimum inhibitory concentrations

The *in vitro* activity of *in silico* identified drugs was evaluated against *C. albicans* ATCC 90028, a strain that shares 99.65% of genomic similarity with SC5314/ATCC MYA-2876 (Benton et al, 2021). Minimum inhibitory concentrations (MIC) of each *in silico* identified drug, as well as FLU and EtBr, were determined using the broth microdilution method, according to the guidelines of the European Committee on Antimicrobial Susceptibility Testing (EUCAST) Definitive Document E.Def 7.4 (European Committee on Antimicrobial Susceptibility Testing (EUCAST), 2023). RPMI-1640 liquid medium (2×) supplemented with 2% glucose was used for all assays. Briefly, two-fold serial dilutions of each drug were prepared in 0.1 mL of RPMI-1640. For drugs soluble in DMSO, the final DMSO concentration in the wells did not exceed 1%. A *C. albicans* suspension equivalent to a McFarland 0.5 standard (BioMérieux, France) was prepared from a 24-hour culture grown in solid medium and, subsequently, diluted 1:10 in sterile deionized water to yield a final inoculum concentration of 0.5–2.5 × 10^5^ CFU/mL. Aliquots of 0.1 mL of this inoculum were added to each well containing the drug dilutions. Positive controls consisted of the fungal suspension in drug-free RPMI-1640 medium both with and without 1% DMSO, while sterile RPMI-1640 medium was included as the negative control. Plates were incubated at 35 °C for 24 hours. Fungal growth was quantified using a Synergy HT fluorometer (Biotek, Santa Clara, USA) by measuring absorbance at 490 nm. The MIC was defined as the lowest drug concentration causing ≥ 50% inhibition of fungal growth compared to the drug-free positive control containing DMSO, following EUCAST recommendations (European Committee on Antimicrobial Susceptibility Testing (EUCAST), 2023). Although the 50% inhibition endpoint is primarily recommended for azole antifungals due to their fungistatic activity and trailing growth effect, frequently observed with this drug class, this threshold was applied uniformly across all tested compounds to enable consistent comparative assessment in the screening assays. Accordingly, the MIC values reported here should be interpreted as comparative measurements within the context of this screening approach rather than as clinical susceptibility determinations. All MIC determinations were performed in three independent experiments, each including two technical replicates.

### Evaluation of the potential adjuvant effect of candidate drugs

To assess the adjuvant effect of candidate drugs on the activity of FLU and EtBr, the MIC_FLU/EtBr_ was determined in the presence and absence of each drug at one-fourth of its MIC. The MIC determination followed the procedure described above, with the exception that two-fold serial dilutions of FLU and EtBr were prepared in 0.05 mL of RPMI-1640, followed by the addition of 0.05 mL of the candidate drug solution. Incubation conditions and result interpretation were performed as previously described. A drug was considered a potential adjuvant if it promoted at least a four-fold reduction in MIC_FLU/EtBr_ (Costa et al, 2016).

### Evaluation of efflux inhibitory activity by real-time fluorometry

The potential efflux inhibitory activity of candidate drugs in *C. albicans* was assessed through real-time fluorometry detection of EtBr accumulation, using an adapted protocol from Rodrigues et al (Rodrigues et al, 2021). To prepare the fungal suspension, one to two colonies of *C. albicans* were inoculated into 5 mL of BHI broth medium and incubated at 37 °C with agitation (180 rpm) for 24 hours. After incubation, fungal cells [(6.55 ± 0.58) × 10^8^ CFU/mL] were harvested by centrifugation at 4.000 rpm for 10 minutes at room temperature. The pellet was washed twice with PBS and resuspended to a final concentration of (1.15 ± 0.60) × 10^9^ CFU/mL. Cell density was quantified through serial dilutions and subsequent plate counting.

Prior to drug testing, the EtBr steady-state concentration (defined as the point at which influx equals efflux) was determined. Serial dilutions of EtBr (0.5 to 16 mg/L) were prepared in PBS, and 0.2 mL microtubes were prepared with 0.05 mL of each EtBr solution and 0.05 mL of fungal suspension, yielding a final volume of 0.1 mL. Fluorescence was measured in real-time over 60 cycles of 1 minute, at 37 °C using The Rotor-Gene™ 3000 system (Corbett Research, Sydney, Australia), with excitation and emission wavelengths set to 530 and 585 nm, respectively.

After establishing the EtBr steady-state concentration (2 mg/L), the effect of candidate drugs on efflux activity in *C. albicans* was evaluated. For a final volume of 0.1 mL, microtubes were prepared containing the fungal suspension, EtBr at 2 mg/L, and the candidate drug at one-fourth of its MIC. The following controls were included: candidate drug in EtBr-free PBS (no cells); candidate drug in EtBr-containing PBS (no cells); EtBr in drug-free PBS (no cells); fungal suspension in EtBr- and drug-free PBS; fungal suspension in EtBr-containing, drug-free PBS. Fluorescence emitted by EtBr was measured using the Rotor-Gene™ 3000 system, under the same conditions described previously.

To quantify efflux inhibition, the Relative Final Fluorescence (RFF) for each drug was calculated based on fluorescence values at minute 60, using the formula:


$$\text{RFF}=\;\left[\text{R}{\text{F}}_{60\;(\text{drug})}–\text{ R}{\text{F}}_{60\;(\text{EtBr})}\right]/\text{R}{\text{F}}_{60\;\left(\text{EtBr}\right),}$$

where RF_60 (drug)_ corresponds to the fluorescence in the presence of both EtBr and the candidate drug, and RF_60 (EtBr)_ represents fluorescence in the presence of EtBr alone. Drugs with RFF ≥ 1 were considered potential efflux inhibitors (Costa et al, 2016).

### Evaluation of synergistic interactions using the checkerboard method

A checkerboard assay was performed to evaluate potential synergistic interactions between FLU or EtBr and candidate drugs that demonstrated potential efflux inhibitory activity (RFF ≥ 1).

Two-fold dilutions were prepared from stock solutions of FLU, EtBr, and each candidate drug to achieve the following concentration ranges: 0.03–16 mg/L for FLU, 2–1024 mg/L for EtBr, and 16–2048 mg/L for the candidate drugs. The highest concentration tested corresponded to four times the MIC of each compound. Assays were performed in 96-well microplates by combining 0.05 mL of FLU or EtBr solution with 0.05 mL of candidate drug solution in each well. The MICs of FLU or EtBr and each candidate drug alone were determined in row H and column 11, respectively. A 0.1 mL aliquot of fungal inoculum was then added to all wells. Column 12 contained controls: four wells with fungal suspension in drug-free RPMI-1640 medium (positive control) and four wells with RPMI-1640 medium alone (negative/sterility control). Plates were incubated at 35 °C for 24 hours, and fungal growth was measured as previously described.

Synergistic interactions were evaluated by calculating the fractional inhibitory concentration index (FICI), using the formula:


$$\begin{array}{c} \text{FICI }=\;\left(\text{MI}{\text{C}}_{\text{EtBr}/\text{FLU }+\text{ drug}/}\text{MI}{\text{C}}_{\text{EtBr}/\text{FLU}}\right)\\ \;+\;\left(\text{MI}{\text{C}}_{\text{drug }+\text{ EtBr}/\text{FLU})/}\text{MI}{\text{C}}_{\text{drug}}\right). \end{array}$$

The interaction was interpreted based on the criteria proposed by Bidaud et al., where a FICI value of ≤ 0.5 indicates synergy, between 0.5 and 4 reveals indifference and > 4 indicates antagonism (Bidaud et al, 2021).

### Statistical analysis

All experiments were performed in independent biological replicates. Data from EtBr accumulation assays are presented as mean ± standard deviation (SD). Due to the exploratory nature of the screening and the limited number of replicates, results were analyzed using descriptive statistics. MICs were determined in independent experiments according to the broth microdilution method. When replicate MIC values differed, the most frequently observed MIC value (modal MIC) was reported.

## Results

**Figure 1:**
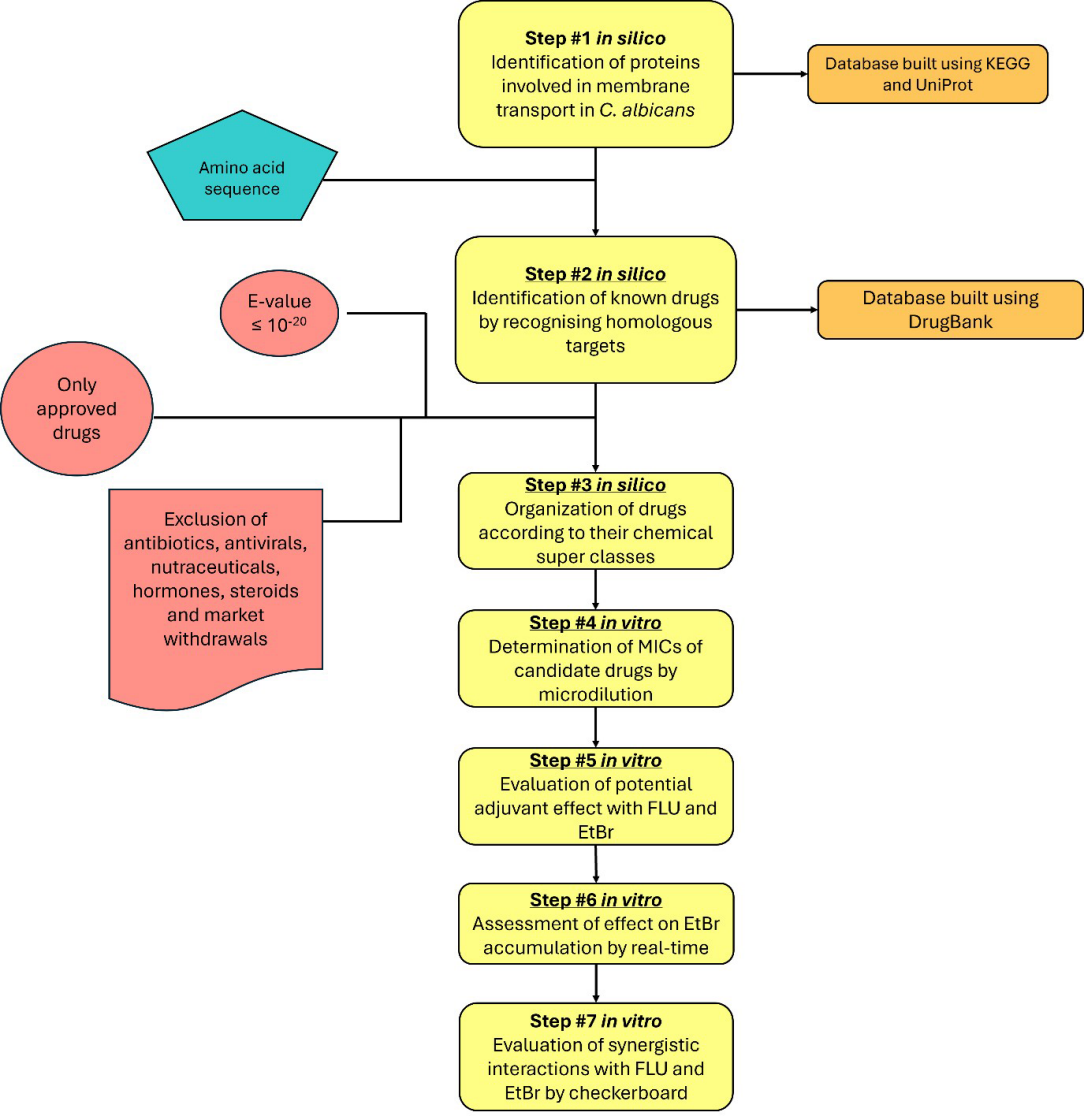
Flowchart summarizing the *in silico* repurposing strategy and subsequent *in vitro* analysis. EtBr, ethidium bromide; FLU, fluconazole; MIC, minimum inhibitory concentration.

The aim of this study was to use an *in silico* drug repurposing strategy to identify approved drugs with potential activity as efflux inhibitors in *C. albicans*. The rationale and workflow of the adopted approach are illustrated in **Figure 1**.

### *In silico* drug repurposing

The *in silico* drug repurposing strategy identified 245 membrane transport proteins in *C. albicans*, 51 of which were associated with 78 homologous targets and 777 approved drugs, after applying exclusion criteria (nutraceuticals, antibiotics, and compounds not suitable for repurposing) (**Supplementary Tables 1**, **2**, **Supplementary Material**).

**Figure 2:**
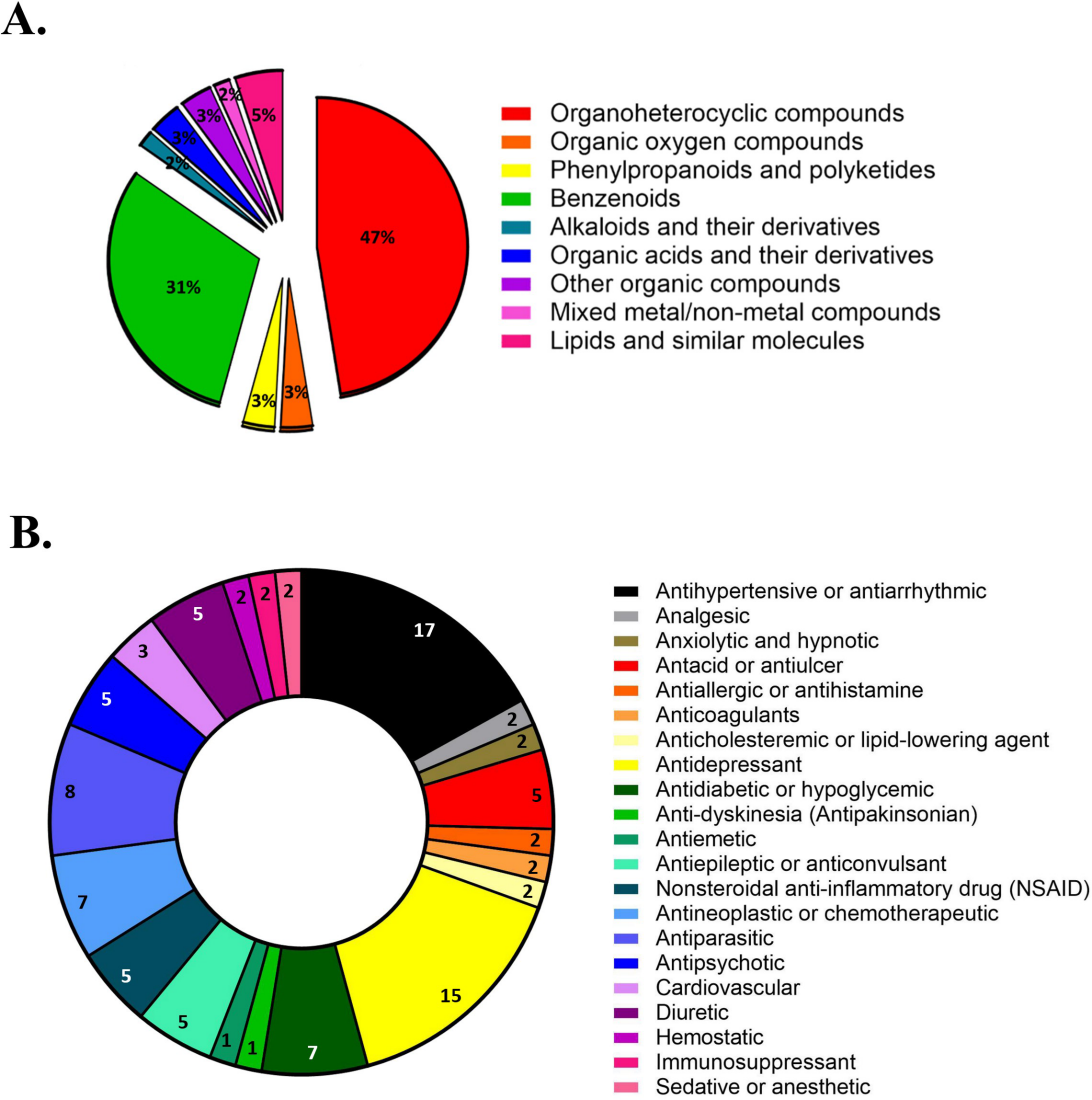
**(A)** Percentage distribution, according to chemical superclass, of the 777 approved drugs retained after applying the exclusion criteria in the *in silico* screening pipeline. **(B)** Percentage distribution of *in silico* identified drugs according to their therapeutical indications.

Among the identified drugs, the most predominant chemical superclass was organic heterocyclic compounds, comprising 28 drugs (47%), followed by benzenoids, with 18 drugs (31%). Superclasses with the fewest representatives included alkaloids and derivatives, and mixed metal/non-metal compounds, each represented by a single drug (**Figure 2A**). To ensure chemical diversity while maintaining experimental feasibility, a subset of 59 drugs was selected for subsequent *in vitro* evaluation by choosing at least one representative compound from each chemical superclass identified among the candidates. When several drugs belonged to the same superclass, selection was prioritized according to their availability, suitability for *in vitro* testing and safety characterization, ensuring their suitability for repurposing strategies. The selected drugs included: acebutolol; albendazole; amlodipine; anastrozole; atenolol; atovaquone; avanafil; azelastine; benzocaine; bisoprolol; buspirone; chloroquine; chlorpromazine; citalopram; desipramine; diltiazem; diosmin; dipyridamole; doxepin; doxorubicin; duloxetine; enalapril; fluoxetine; flurbiprofen; fluvoxamine; gliquidone; hydrochlorothiazide; imipramine; istradefylline; ketoprofen; lansoprazole; lovastatin; meloxicam; metformin; metoclopramide; miltefosine; mycophenolate mofetil; nifedipine; nizatidine; olanzapine; phenytoin; probenecid; procainamide; ranitidine; repaglinide; reserpine; risperidone; sertraline; streptozocin; sulfinpyrazone; sulpiride; sunitinib; tinidazole; tolbutamide; topiramate; valproic acid; valsartan; verapamil; and zolmitriptan. The selected drugs were also categorized according to their therapeutic classes, with 21 distinct pharmacological classes identified, with antihypertensives/antiarrhythmics (17%) and antidepressants (15%) being the most prevalent (**Figure 2B**).

### *In vitro* evaluation of candidate drugs

From the 59 candidate drugs selected for *in vitro* analysis, 39 compounds (66%) presented MICs equal to or above 1024 mg/L, while the remaining drugs showed MICs ranging from 2 to 512 mg/L. Miltefosine demonstrated the highest antifungal activity, with the lowest MIC of 2 mg/L (**Table 1**). Regarding the potential adjuvant effect of the tested drugs on FLU activity, only procainamide and amlodipine promoted a four- and eight-fold reduction in the MIC_FLU_ (from 0.25 to 0.06 mg/L and 0.03 mg/L, respectively). No additional drugs produced a ≥ four-fold reduction in MIC_FLU_.

**Table 1:** MICs values of *in silico* identified drugs tested against *C. albicans* ATCC 90028.

Drugs	MIC (mg/L)
MIL	2
SER	32
CHL; MYC	64
SUN	128
AML; AZE; DOXO; DUL; FUO; LAN; NIF	256
BEN; DES; DOX; FLV; IMI; ISF; RES; RIS	512
ACE; ALB; ANA; AVA; BUS; CIT; DIP; FLR; GLI; HYD; KET; LOV; MEL; MET; NIZ; OLA; PHE; PBC; PRO; RAN; REP; SPZ; SUL; TINI; TOL; TOP; VAL; VST; VP; ZOL	1024
ATE; ATV; BIS; CLO; DIL; DIO; ENA; METO; STREP	> 1024

ACE, Acebutolol; ALB, Albendazole; AML, Amlodipine; ANA, Anastrozole; ATE, Atenolol; ATV, Atovaquone; AVA, Avanafil; AZE, Azelastine; BEN, Benzocaine; BIS, Bisoprolol; BUS, Buspirone; CLO, Chloroquine; CHL, Chlorpromazine; CIT, Citalopram; DES, Desipramine; DIL, Diltiazem; DIO, Diosmin; DIP, Dipyridamole; DOX, Doxepin; DOXO, Doxorubicin; DUL, Duloxetine; ENA, Enalapril; FUO, Fluoxetine; FLR, Flurbiprofen; FLV, Fluvoxamine; GLI Gliquidone; HYD, Hydrochlorothiazide; IMI, Imipramine; ISF, Istradefylline; KET, Ketoprofen; LAN, Lansoprazole; LOV, Lovastatin; MEL, Meloxicam; MET, Metformin; METO, Metoclopramide; MIL, Miltefosine; MYC, Mycophenolate mofetil; NIF, Nifedipine; NIZ, Nizatidine; OLA, Olanzapine; PHE, Phenytoin; PBC, Probenecid; PRO, Procainamide; RAN, Ranitidine; REP, Repaglinide; RES, Reserpine; RIS, Risperidone; SER, Sertraline; STREP, Streptozocin; SPZ, Sulfinpyrazone; SUL, Sulpiride; SUN, Sunitinib; TINI, Tinidazole; TOL, Tolbutamide; TOP, Topiramate; VAL, Valproic acid; VST, Valsartan; VP, Verapamil; ZOL, Zolmitriptan; MIC, Minimum Inhibitory Concentration.

The effect of the selected drugs on MIC_EtBr_ was also determined. Thirteen drugs (avanafil, chloroquine, fluoxetine, ketoprofen, istradefylline, metoclopramide, miltefosine, olanzapine, sertraline, amlodipine, fluvoxamine, lansoprazole, and sunitinib) demonstrated potential as EtBr adjuvants, reducing MIC_EtBr_ by four-, eight-, and sixteen-fold. The greatest reduction was observed for sunitinib, which decreased MIC_EtBr_ from 64 to 4 mg/L (**Table 2**).

**Table 2:** Candidate drugs that promoted a reduction in the MIC value (mg/L) of EtBr against *C. albicans* ATCC 90028.

Drugs	MIC_EtBr_
–	64
AVA; CHL; FUO; KET; ISF; METO; MIL; OLA; SER	16 (↓4×)
AML; FLV; LAN	8 (↓8×)
SUN	4 (↓16×)

Candidate drugs were used at one-fourth their MIC values. AML, Amlodipine; AVA, Avanafil; CHL, Chlorpromazine; EtBr, ethidium bromide; FUO, Fluoxetine; FLV, Fluvoxamine; KET, Ketoprofen; ISF, Istradefylline; LAN, Lansoprazole; METO, Metoclopramide; MIC, minimum inhibitory concentration; MIL, Miltefosine; OLA, Olanzapine; SER, Sertraline; SUN, Sunitinib.↓ indicates fold reduction in MIC relative to the value in the absence of drug.

**Figure 3:**
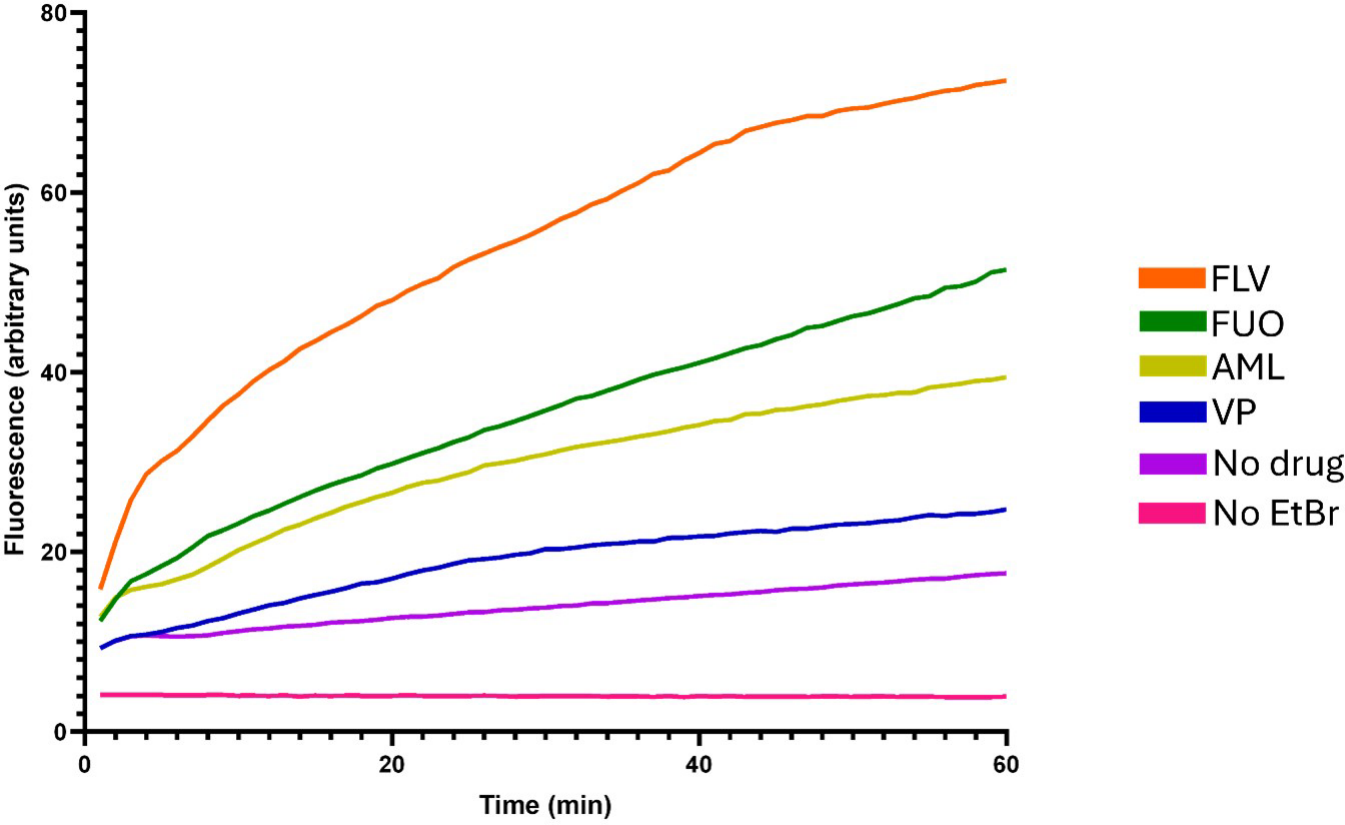
EtBr accumulation in *C. albicans* in the absence and presence of candidate drugs. EtBr was used at 2 mg/L (steady-state concentration) and the tested drugs were used at one-fourth their respective MIC values. The results represent the mean of three independent experiments. AML, amlodipine; EtBr, ethidium bromide; FLU, fluconazole; FLV, fluvoxamine; FUO, fluoxetine; VP, verapamil.

Subsequently, the candidate drugs were evaluated for efflux inhibitory activity in *C. albicans* ATCC 90028, using a real-time fluorometric assay. EtBr, a common efflux pump substrate, was used as a marker for efflux activity, since its fluorescence increases with intracellular accumulation (Costa et al, 2016; Rodrigues et al, 2021). Due to technical limitations related to fluorescence spectrum overlap, doxorubicin and gliquidone were excluded from these assays. As shown in **Figure 3**, fluvoxamine, fluoxetine and amlodipine promoted greater EtBr accumulation than the reference efflux inhibitor verapamil. These results were corroborated by their RFF values, with fluvoxamine, fluoxetine and amlodipine presenting an RFF ≥ 1, classifying them as potential efflux inhibitors. In contrast, verapamil exhibited a lower RFF, indicating milder activity, and the remaining drugs showed no effect in EtBr accumulation (**Table 3**, **Supplementary Table 3**, **Supplementary Material**).

**Table 3:** RFF values obtained for candidate drugs that promoted EtBr accumulation in *C. albicans* ATCC 90028.

Drugs	RFF ± SD
FLV	3.00 ± 0.33
AML	1.56 ± 0.47
FUO	1.24 ± 0.05
VP	0.59 ± 0.26

RFF values reflect relative EtBr accumulation under the tested conditions and were calculated based on fluorescence values at minute 60. Data presented correspond to the average of three independent assays. AML, Amlodipine; FLV, Fluvoxamine; FUO, Fluoxetine; RFF, Relative Final Fluorescence; SD, Standard Deviation; VP, Verapamil. Candidate drugs were used at one-fourth their MIC values.

To further investigate their potential interactions, the drugs that showed the most promising results in the EtBr accumulation assays (fluvoxamine, fluoxetine, amlodipine and verapamil) were tested in combination with FLU or EtBr, against *C. albicans* ATCC 90028, using the checkerboard method. Amlodipine showed no interaction with FLU (FICI values between 0.5 and 4), while fluvoxamine, fluoxetine and verapamil displayed an antagonistic effect (FICI > 4) with this antifungal agent. In combination with EtBr, amlodipine and fluvoxamine showed synergistic interactions with EtBr, with FICI values ≤ 0.5, whereas fluoxetine and verapamil exhibited no interaction (**Table 4**).

**Table 4:** FICI values for the interaction between FLU or EtBr and the candidate drugs amlodipine, fluoxetine, fluvoxamine, and verapamil against *C. albicans* ATCC 90028.

Drugs	FICI_FLU_ ± SD	FICI_EtBr_ ± SD
AML	2.61 ± 0.60	0.33 ± 0.06
FLV	8.33 ± 0.12	0.42 ± 0.06
FUO	8.08 ± 0.03	0.54 ± 0.06
VP	4.02 ± 0.01	1.35 ± 0,47

AML, Amlodipine; EtBr, ethidium bromide; FICI, Fractional Inhibitory Concentration Index; FLU, Fluconazole; FLV, Fluvoxamine; FUO, Fluoxetine; VP, Verapamil.

## Discussion

*C. albicans* is one of the most prevalent and clinically significant fungal pathogens worldwide. The burden of infections caused by *C. albicans* has been exacerbated by the increasing incidence of antimicrobial resistance, particularly to azoles, the most commonly used antifungal agents in the treatment of candidiasis (Arastehfar et al, 2020). Given the limited arsenal of currently available antifungals and the time-consuming nature of *de novo* drug development, drug repurposing has emerged as a promising strategy to rapidly identify alternative therapeutic options to combat antimicrobial resistance (Rodrigues et al, 2020; Vaitkienė et al, 2020; Ganeshkumar et al, 2021; Kulkarni et al, 2023).

In this study, we applied a chemogenomics-based drug repurposing strategy, previously used to identify compounds with activity against several pathogens, including *Schistosoma mansoni* (Neves et al, 2015), *Mycobacterium tuberculosis* (Rodrigues et al, 2020), and SARS-CoV-2 (Rodrigues et al, 2022), among others (de Oliveira et al, 2019), as a phenotypic screening approach to identify approved drugs capable of modulating efflux-related mechanisms in *C. albicans*. Rather than aiming to validate specific transporter-drug interactions, this work explored whether drugs predicted to be associated with membrane transport functions could induce functional efflux inhibition as reflected by increased intracellular accumulation of efflux substrates. This strategy enables the identification of candidate efflux inhibitors independently of prior assumptions regarding transporter specificity or resistance phenotype.

To support this approach, two databases were used to compile a comprehensive list of membrane transport proteins in *C. albicans*, with a particular focus on efflux pump systems. This analysis resulted in the identification of 51 potential transport-related targets, including several subfamilies of the ABC and MFS transporters, which represent two of the most prominent efflux systems implicated in azole resistance in *C. albicans* (Prasad and Singh, 2013; Prasad and Rawal, 2014). Subsequent homology-based searches identified 777 approved drugs potentially associated with these transport proteins. Several of these compounds have previously been explored in antifungal drug repurposing studies, including curcumin (Lee et al, 2022), verapamil (Vega-Chacón et al, 2021), reserpine (Clark et al, 1996), and lovastatin (Zhou et al, 2018). To ensure chemical diversity while maintaining experimental feasibility, a subset of 59 drugs was selected for *in vitro* evaluation, with at least one compound from each chemical superclass.

Among the selected compounds, antihypertensives/antiar-rhythmics and antidepressants were the most prevalent pharmacological classes. This observation is consistent with previous studies demonstrating that drugs such as immunosuppressants, antipsychotics, antidepressants, non-steroidal anti-inflammatory drugs, and antiarrhythmics hold potential for antifungal repurposing, showing promising results in both *in vitro* and *in vivo* models, as well as synergistic effects when combined with conventional antifungal agents (Miró-Canturri et al, 2019; Zhang et al, 2021; Izadi et al, 2022). Regarding the chemical superclass, organoheterocyclic compounds and benzenoids were the most frequently represented among the drugs identified *in silico*. Notably, clinically used antifungals such as itraconazole belong to the organoheterocyclic superclass, whereas voriconazole and FLU are classified as benzenoid compounds.

From the set of 59 drugs evaluated *in vitro*, the antileishmanial agent miltefosine exhibited the lowest MIC (IC_50_) of 2 mg/L. This finding is consistent with previous reports demonstrating *in vitro* activity of miltefosine against *C. albicans*, with MIC_90_ values ranging from 1 to 2 mg/L for both reference strains and clinical isolates (Vila et al, 2015). Similarly, Wu et al. reported that miltefosine is active against azole-resistant *Candida* spp., with a mean MIC value of 2 mg/L and proposed that its antifungal activity involves intracellular reactive oxygen species generation and induction of apoptosis (Wu et al, 2023). In addition, miltefosine has demonstrated activity against azole-resistant *Aspergillus* strains and multiple *Mucorales* species, including biofilm-forming isolates (Haghani et al, 2023; Xisto et al, 2023). Importantly, the antifungal activity of miltefosine appears to be independent of efflux inhibition, as its efficacy is retained in azole-resistant isolates, suggesting a potentially distinct mechanism of action.

Fluvoxamine, fluoxetine and amlodipine demonstrated potential efflux inhibitory activity in *C. albicans*, as indicated by increased intracellular EtBr accumulation, while verapamil also promoted EtBr accumulation, although to a lesser extent. EtBr was employed as a surrogate substrate to monitor efflux activity due to its broad recognition by multiple fungal multidrug transporters and has previously been used to assess efflux activity in fungi (Passos Lima et al., 2025). However, the increase in intracellular fluorescence observed in EtBr accumulation assays may also be influenced by factors other than efflux inhibition, including alterations in membrane permeability or broader changes in cellular physiology and stress responses. Therefore, although the experimental design included controls to minimize these effects, the EtBr assay provides only an indirect measure of efflux modulation and does not allow discrimination between specific transporter inhibition and other mechanisms affecting dye uptake. Consequently, the use of a single fluorochrome represents a limitation to this approach. Future studies employing additional efflux substrates, such as rhodamine 123, as well as transporter specific or genetic approaches, will be necessary to further clarify the mechanisms underlying the observed efflux modulating phenotypes.

Although several compounds promoted increased intracellular accumulation of EtBr, suggesting potential efflux inhibitory activity, this effect did not consistently translate into synergistic interactions with fluconazole. Azole susceptibility in *C. albicans* is determined by multiple mechanisms beyond drug efflux, including alterations in ergosterol biosynthesis, target enzyme modifications, and activation of cellular stress responses. Consequently, inhibition of efflux transporters alone may not be sufficient to produce measurable synergy with fluconazole in checkerboard assays. In addition, the EtBr accumulation assay provides an indirect measure of efflux modulation and reflects the activity of multiple transport systems, some of which may not play a major role in fluconazole extrusion. This may explain why compounds capable of increasing EtBr accumulation do not necessarily enhance fluconazole susceptibility. Notably, amlodipine reduced the MIC of fluconazole in combination assays. However, this interaction did not meet the criteria for synergy in the checkerboard analysis. These observations highlight the complexity of antifungal drug interactions and suggest that efflux modulation may influence azole activity without necessarily producing strong synergistic effects under the experimental conditions tested.

Verapamil, a non-dihydropyridine calcium channel blocker used in the treatment of angina, arrhythmia, and hypertension (Wishart et al, 2018; Shiozaki et al, 2021), has previously been reported to inhibit efflux pumps in *C. albicans*. In particular, Vega-Chacón et al. demonstrated verapamil mediated efflux inhibition using rhodamine 123 accumulation assay and reported synergistic interactions with FLU, reduced biofilm formation and increased survival of *Galleria mellonella* larvae infected with *C. albicans* (Vega-Chacón et al, 2021). In contrast, under the experimental conditions used in the present study, antagonistic interactions between verapamil and FLU were observed.

Amlodipine, a dihydropyridine calcium channel blocker used to treat hypertension and angina (Wishart et al, 2018; Sheraz et al, 2016), also demonstrated a potential adjuvant effect on FLU activity, reducing its MIC by eight-fold. Previous studies have reported MIC values of 256 mg/L for amlodipine against *C. albicans*, consistent with the results observed here, and have shown activity against both planktonic and biofilm forms of *Candida glabrata* and *C. albicans* (Gupta et al, 2016). Additional studies have reported MICs ranging from 62.5 to 250 mg/L against *Candida* spp., along with notable anti-biofilm effects and induction of apoptosis (Queiroz et al, 2024). Beyond its antifungal activity, amlodipine also demonstrated antimicrobial activity against bacterial pathogens, including *Staphylococcus aureus*, both *in vitro* and in invertebrate infection models (Boyd et al, 2021; Andrade et al, 2025).

Fluoxetine, a selective serotonin reuptake inhibitor indicated for the treatment of major depressive disorder, obsessive compulsive disorder, and bulimia nervosa, has also been reported to exhibit antimicrobial activity. Previous studies evaluating fluoxetine alone and in combination with FLU against *C. albicans* reference strains and clinical isolates reported no interaction between the two drugs (Oliveira et al., 2014; Jiang et al, 2020), contrasting with the antagonistic effect observed in the present study under the tested conditions. In addition, fluoxetine has demonstrated activity against multidrug-resistant bacterial strains, including *Pseudomonas aeruginosa*, *S. aureus* and *Escherichia coli*, highlighting its broader antimicrobial potential (Karine de Sousa et al, 2018).

To our knowledge, this study represents the first report describing the efflux inhibitory potential of fluvoxamine, a selective serotonin-reuptake inhibitor prescribed for the treatment of obsessive-compulsive disorder (Altamura et al, 2015; Wishart et al, 2018), in *C. albicans*. Previous work evaluating fluvoxamine against *C. glabrata*, reported MIC values ranging from 62.5 to 250 mg/L and an indifferent interaction with FLU in checkerboard assays (Alkhalifa et al, 2022). In addition, fluvoxamine has attracted attention for its potential activity against SARS-CoV-2 (Bramante et al, 2022; Mahdi et al, 2022; Reis et al, 2023; Tsiakalos et al, 2023; Prasanth et al, 2024).

It is important to note that the efflux inhibitory effects observed for several compounds occurred at concentrations that may exceed clinically achievable plasma levels. This limitation is common to many efflux inhibitors described to date, including classical inhibitors such as verapamil and reserpine. In this context, the relevance of the present findings lies not in the immediate clinical repurposing of these drugs, but rather in their potential role as chemical scaffolds or mechanistic probes for the development of more potent and selective efflux inhibitors.

Although several of the identified compounds are clinically approved drugs with established safety profiles, the concentrations required to observe efflux modulation or antifungal interactions *in vitro* may exceed clinically achievable plasma levels and may not directly reflect therapeutically relevant exposure. Therefore, the compounds identified here should primarily be regarded as potential chemical scaffolds or mechanistic probes for the modulation of fungal transport systems rather than immediately repurposable therapeutics. Further studies evaluating cytotoxicity and selectivity in mammalian cell models will be necessary to determine the therapeutic window and to assess the potential of these compounds as antifungal adjuvants. Nevertheless, these findings provide a useful starting point for the identification or optimization of molecules capable of targeting efflux-mediated antifungal tolerance.

Another limitation of this study is that the *in vitro* evaluation of candidate drugs was conducted using a single azole-susceptible *C. albicans* reference strain (ATCC 90028). Although this strain is widely used as a quality control strain in antifungal susceptibility testing and provides a reproducible and well characterized model system, it does not fully capture the clinical complexity and genetic diversity associated with efflux-mediated azole resistance. Nevertheless, the use of a susceptible reference strain allowed the identification of efflux modulating phenotypes independently of pre-existing resistance mechanisms, minimizing potential confounding effects related to strain-specific mutations or differential expression of resistance determinants. Therefore, this approach supports the use of this model as an initial screening platform to detect compounds capable of modulating transport activity. However, responses to efflux modulators may vary among clinical isolates due to differences in genetic background and resistance mechanisms. Future studies should extend these findings to a broader panel of clinical isolates, particularly azole-resistant strains exhibiting documented overexpression of efflux pump genes, in order to assess the clinical relevance and general applicability of the identified compounds.

The antagonistic interactions observed between FLU and certain candidate drugs, including verapamil and fluoxetine, contrast with previous reports describing synergistic or indifferent effects. These discrepancies may be explained by important methodological and biological differences between studies. Previous reports demonstrating synergism were frequently conducted using fluconazole-resistant isolates or biofilm models, where efflux pump overexpression and reduced azole susceptibility are more prominent (Oliveira et al., 2014; Jiang et al., 2020). In addition, the study by Vega-Chacón et al. (2021) combined checkerboard assays with alternative analytical approaches, such as Bliss independence, and further validated drug interactions in biofilm and *in vivo* infection models, which may influence the classification and interpretation of drug interactions. In contrast, the present study was performed using the azole-susceptible reference strain *C. albicans* ATCC 90028 under planktonic growth conditions, where efflux-mediated resistance mechanisms are less pronounced and may have a reduced impact on fluconazole activity. Furthermore, differences in experimental parameters, including growth medium composition, inoculum size, drug concentration ranges, incubation time, and susceptibility endpoints used to determine FICI values, may significantly affect the outcome of checkerboard assays. Importantly, inhibition of efflux activity does not necessarily translate into antifungal synergy, as drug-drug interactions may involve complex cellular processes beyond transporter modulation. FLU exerts its antifungal activity by inhibiting Erg11, a key enzyme in the ergosterol biosynthesis pathway, thereby disrupting membrane integrity and function. Compounds that alter membrane composition, sterol homeostasis, or cellular stress responses may therefore modulate the cellular effects of FLU. For example, changes in membrane fluidity or lipid organization could influence drug uptake or intracellular distribution, while activation of adaptive stress pathways may promote cellular tolerance to azole-induced stress (Kohli et al, 2002). Taken together, these findings highlight the complexity of antifungal drug interactions in *C. albicans* and suggest that the antagonistic effects observed in this study may arise from indirect physiological adaptations rather than direct interference with the azole target pathway.

In summary, this study demonstrates that a homology-driven drug repurposing strategy combined with functional *in vitro* assays constitutes an efficient platform for the identification of candidate efflux inhibitors in *C. albicans*. Although the compounds identified did not consistently display synergistic interactions with fluconazole under the tested conditions, fluvoxamine, fluoxetine and amlodipine exhibited reproducible efflux inhibitory activity. These findings support further investigation of efflux inhibition as a complementary strategy to overcome antifungal resistance and highlight the value of drug repurposing approaches in expanding the antifungal discovery pipeline.

## Data Availability

The original contributions presented in this study are included in the article/**Supplementary Material**. The datasets analyzed were retrieved from publicly available databases, including KEGG, UniProt, and DrugBank. No new datasets were generated or deposited in external repositories. Further inquiries can be directed to the corresponding author.
